# Supplementary material for: Sociality genes are associated with human-directed social behaviour in golden and Labrador retriever dogs
Source: PeerJ. 2018 Nov 6;6:e5889. doi: 10.7717/peerj.5889 (PMC6225837; doi:10.7717/peerj.5889)
Supplement: Supplemental Information 1 [file peerj-06-5889-s001.docx]

Supplementary Table S1: Detailed information about the Scandinavian wolf samples

| **Sample ID** | **Sex** | **House name** | **Born** | **Sire** | **Dam** | **Birthplace** | **Sample type** | **Sample date** | **Donated from** |
| --- | --- | --- | --- | --- | --- | --- | --- | --- | --- |
| 18 | M | Snipe | 2003 | T579 | T747 | Skåne | Blood | 09-09-22 | Kolmården Wildlife Park |
| 15 | M | Ludo | 2003 | T564 | T675 | Skåne | Blood | 11-03-23 | Kolmården Wildlife Park |
| 17 | M | Moody | 2003 | T564 | T675 | Skåne | Blood | 11-03-23 | Kolmården Wildlife Park |
| 7 | F | Miraz | 2007 | T740 | T757 | Lycksele | Serum | 08-02-15 | Kolmården Wildlife Park |
| 2 | M | Tash | 2007 | T641 | T825 | Borås | Serum | 08-02-15 | Kolmården Wildlife Park |
| 9 | F | Lucy | 2007 | T641 | T825 | Borås | Blood | 08-02-15 | Kolmården Wildlife Park |
| 5 | F | Jadis | 2007 | T641 | T825 | Borås | Blood | 08-02-15 | Kolmården Wildlife Park |
| 3 | F | Narnia | 2007 | T641 | T825 | Borås | Blood | 08-02-15 | Kolmården Wildlife Park |
| 1 | M | Aslan | 2007 | T786 | T796 | Langedrag | Serum | 08-02-15 | Kolmården Wildlife Park |
| 11 | M | Aravis | 2007 | T786 | T796 | Langedrag | Blood | 08-02-15 | Kolmården Wildlife Park |
| 16 | M | Farkas | 2009 | T641 | T825 | Borås | Blood | 09-11-13 | Kolmården Wildlife Park |
| 12 | M | Vilkas | 2009 | T641 | T825 | Borås | Blood | 09-11-13 | Kolmården Wildlife Park |
| 13 | M | Ulfur | 2009 | T641 | T825 | Borås | Blood | 09-11-13 | Kolmården Wildlife Park |
| 4 | M | Tchono | 2009 | T910 | T896 | Nordens Ark | Blood | 09-11-13 | Kolmården Wildlife Park |
| 10 | M | Amaroq | 2009 | T910 | T896 | Nordens Ark | Blood | 16-01-23 | Kolmården Wildlife Park |
| 8 | M | Lobo | 2009 | T795 | T872 | Skåne | Blood | 09-11-13 | Kolmården Wildlife Park |
| 6 | M | Volk | 2009 | T795 | T872 | Skåne | Blood | 09-11-13 | Kolmården Wildlife Park |
| 14 | M | Kurt | 2009 | T795 | T872 | Skåne | Blood | 09-11-13 | Kolmården Wildlife Park |
| 130 | F | Beata | 2013 | Aslan | Usne | Borås | Brain | 15-11-05 | Borås Animal Park |
| 136 | F | Usne | 2006 |  |  | Riga, Latvia | Brain | 15-11-05 | Borås Animal Park |
| 120 | F | Bibbi | 2013 | Aslan | Usne | Borås | Brain | 15-11-05 | Borås Animal Park |
