# Supplementary material for: Sociality genes are associated with human-directed social behaviour in golden and Labrador retriever dogs
Source: PeerJ. 2018 Nov 6;6:e5889. doi: 10.7717/peerj.5889 (PMC6225837; doi:10.7717/peerj.5889)
Supplement: Supplemental Information 2 — In cases of few observations of a coded behaviour the correlation values are replaced with “NA”. [file peerj-06-5889-s002.docx]

Supplementary Table S2. Inter-observer correlations of 10% of the individuals coded with the unsolvable task. In cases of to few observations of a coded behaviour the correlation values are replaced with “NA”.

| **Breed** | **Behaviour** | **N** | **Spearman** | **P (Spearman)** | **Pearson** | **P (Pearson)** |
| --- | --- | --- | --- | --- | --- | --- |
| Labrador | Frequency experimenter zone | 10 | 0,909 | <0,001 | 0,945 | <0,001 |
| Labrador | Duration experimenter zone | 10 | 0,905 | <0,001 | 0,92 | <0,001 |
| Labrador | Frequency owner zone | 10 | 0,803 | 0,005 | 0,901 | <0,001 |
| Labrador | Duration owner zone | 10 | 0,821 | 0,004 | 0,948 | <0,001 |
| Labrador | Frequency experimenter gaze | 10 | 0,92 | <0,001 | 0,969 | <0,001 |
| Labrador | Duration experimenter gaze | 10 | 0,915 | <0,001 | 0,916 | <0,001 |
| Labrador | Frequency owner gaze | 10 | 0,972 | <0,001 | 0,968 | <0,001 |
| Labrador | Duration owner gaze | 10 | 0,948 | <0,001 | 0,981 | <0,001 |
| Labrador | Frequency physical contact experimenter | 10 | 0,99 | <0,001 | 0,983 | <0,001 |
| Labrador | Duration physical contact experimenter | 10 | 1 | <0,001 | 0,993 | <0,001 |
| Labrador | Frequency physical contact owner | 10 | NA | NA | NA | NA |
| Labrador | Duration physical contact owner | 10 | NA | NA | NA | NA |
| Golden | Frequency experimenter zone | 6 | 0,925 | 0,008 | 0,964 | 0,002 |
| Golden | Duration experimenter zone | 6 | 0,943 | 0,005 | 0,942 | 0,005 |
| Golden | Frequency owner zone | 6 | 0,925 | 0,008 | 0,99 | <0,001 |
| Golden | Duration owner zone | 6 | 1 | <0,001 | 0,976 | 0,001 |
| Golden | Frequency experimenter gaze | 6 | 0,868 | 0,008 | 0,943 | 0,005 |
| Golden | Duration experimenter gaze | 6 | 0,829 | 0,042 | 0,993 | <0,001 |
| Golden | Frequency owner gaze | 6 | 0,868 | 0,025 | 0,985 | <0,001 |
| Golden | Duration owner gaze | 6 | 1 | <0,001 | 0,999 | <0,001 |
| Golden | Frequency physical contact experimenter | 6 | NA | NA | NA | NA |
| Golden | Duration physical contact experimenter | 6 | NA | NA | NA | NA |
| Golden | Frequency physical contact owner | 6 | NA | NA | NA | NA |
| Golden | Duration physical contact owner | 6 | NA | NA | NA | NA |
