# Supplementary material for: Sociality genes are associated with human-directed social behaviour in golden and Labrador retriever dogs
Source: PeerJ. 2018 Nov 6;6:e5889. doi: 10.7717/peerj.5889 (PMC6225837; doi:10.7717/peerj.5889)

Supplementary FigureS1: figures of all genotype-behaviour associations for both golden and Labrador retrievers. Page 2 shows SNP1 and experimenter-directed interactions; page 3 shows SNP1 and owner-directed interactions; page 4 shows SNP2 and experimenter-directed interactions; page 5 shows SNP2 and owner-directed interactions.

SNP1 (BICF2G630798942)

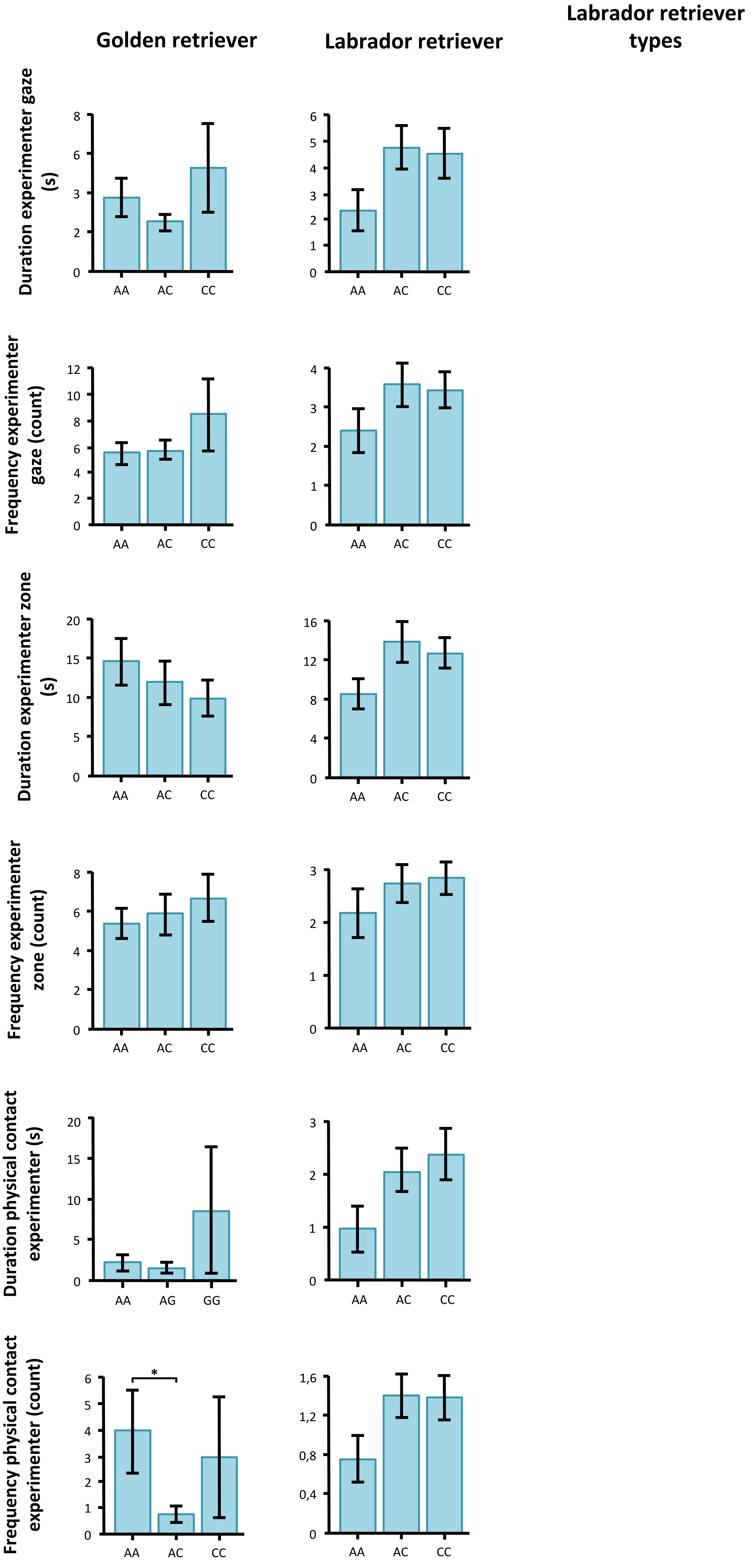

SNP1 (BICF2G630798942)

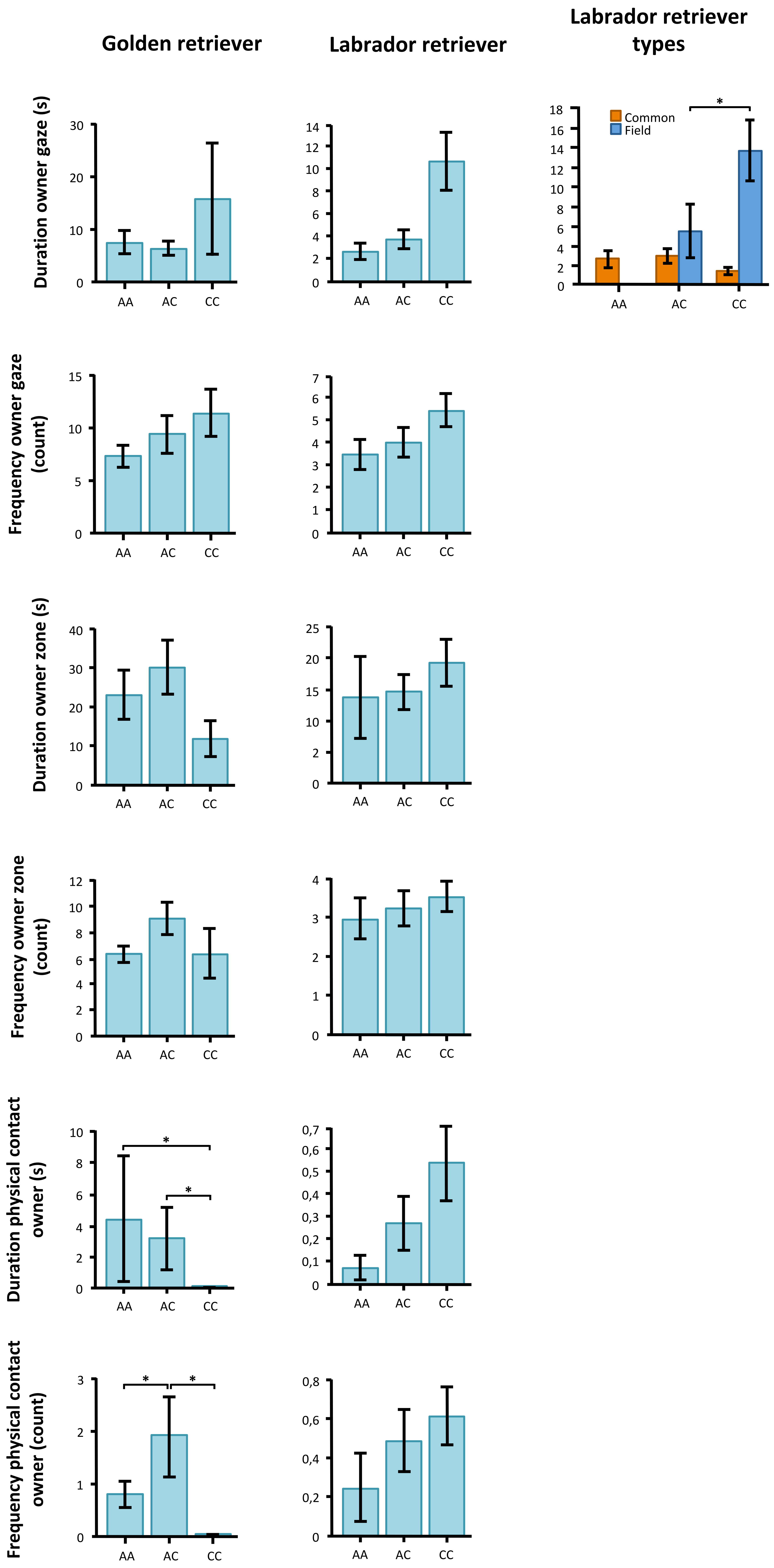

SNP2 (BICF2S23712114)

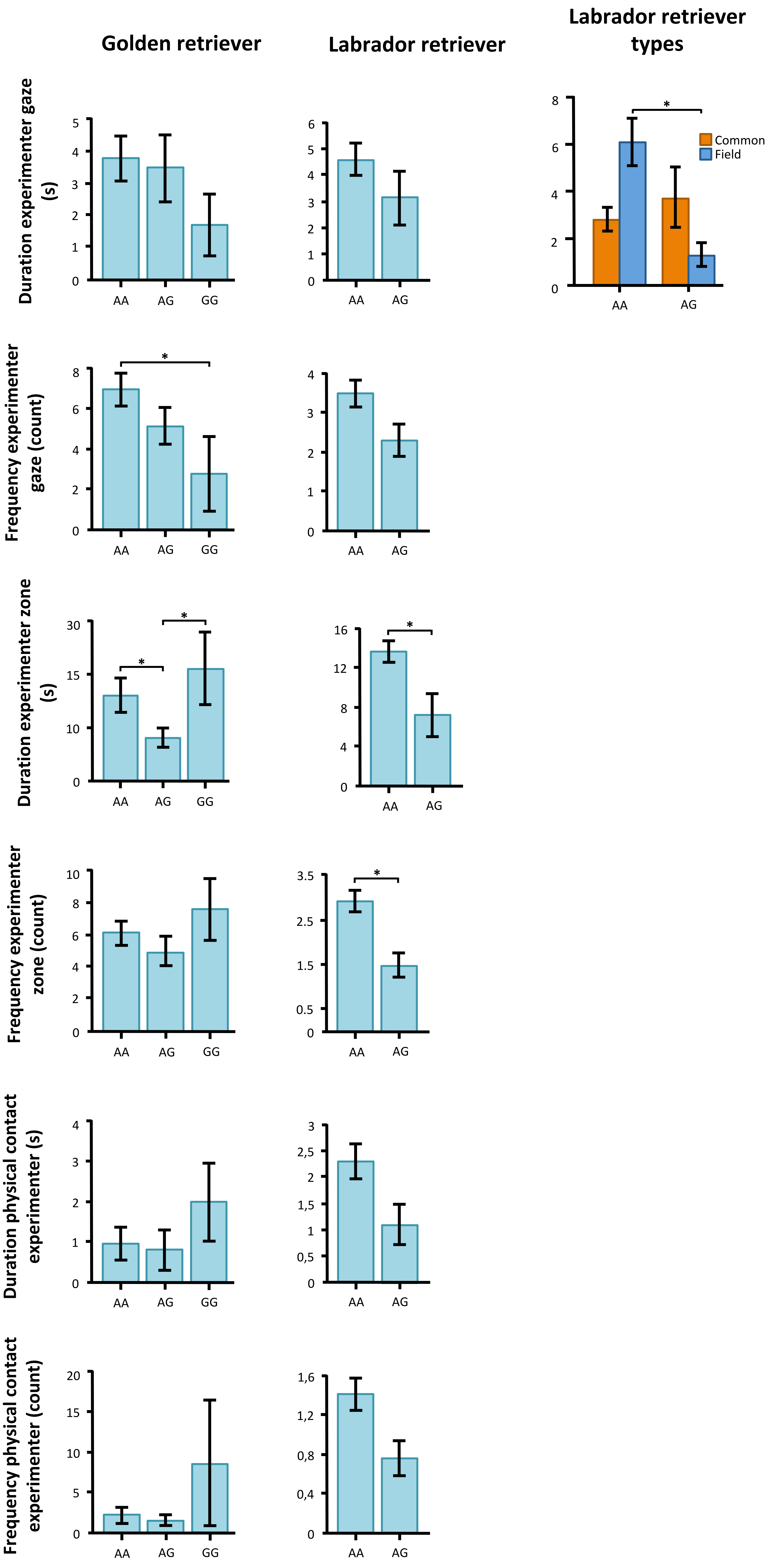

SNP2 (BICF2S23712114)

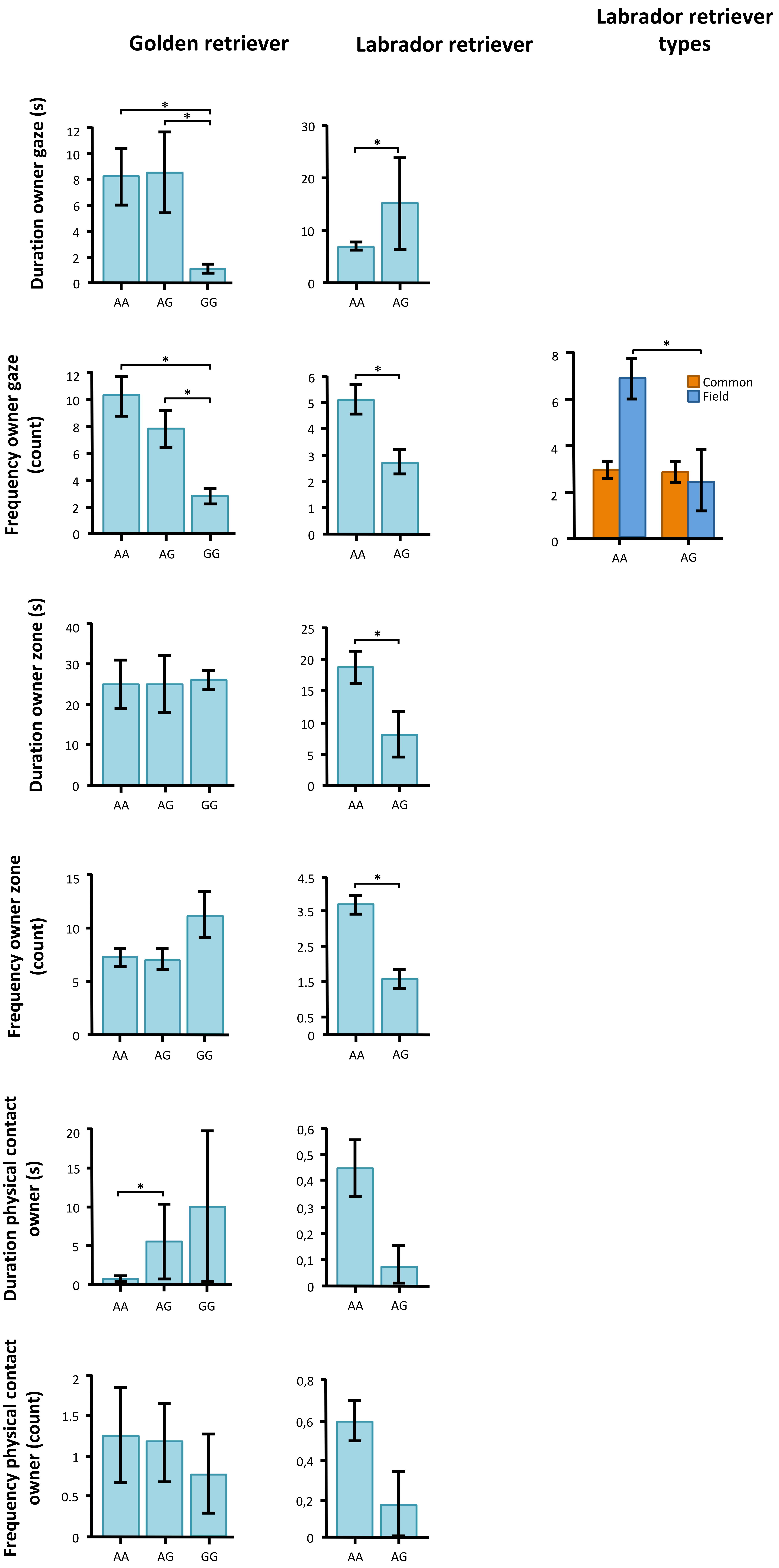

Supplement: Supplemental Information 3 — Page 2 shows SNP1 and experimenter-directed interactions; page 3 shows SNP1 and owner-directed interactions; page 4 shows SNP2 and experimenter-directed interactions; page 5 shows SNP2 and owner-directed interactions. [file peerj-06-5889-s003.pdf]
